# Impacts of multitrophic biological loop on soil carbon sequestration

**DOI:** 10.1093/nsr/nwag475

**Published:** 2026-08-06

**Authors:** Guixiang Zhou, Junxi Liu, Jiabao Zhang

**Affiliations:** State Key Laboratory of Soil and Sustainable Agriculture, Institute of Soil Science, Chinese Academy of Sciences, China; State Key Laboratory of Soil and Sustainable Agriculture, Institute of Soil Science, Chinese Academy of Sciences, China; University of Chinese Academy of Sciences, China; State Key Laboratory of Soil and Sustainable Agriculture, Institute of Soil Science, Chinese Academy of Sciences, China

## Abstract

Interactions among soil viruses, microbes and soil fauna form a unified soil biological loop that shapes microbial necromass and controls soil carbon storage. This work extends the microbial carbon pump theory by introducing the virus-mediated microbial carbon pump, offering a new framework to explain soil carbon sequestration under global change.

Soil organic matter (SOM) represents the largest reservoir of terrestrial organic carbon, and its sustainable management is crucial for mitigating rising atmospheric CO₂ levels. The turnover of SOM is increasingly recognized as being driven by biota communities, ranging from low trophic microorganisms to high trophic fauna. The soil food web mainly depicts trophic interactions, predation and energy flow among soil organisms. The microbial loop, originally proposed for marine ecosystems, refers to a trophic pathway in which dissolved organic matter (DOM) is incorporated into bacterial biomass and subsequently transferred through flagellates and microzooplankton before carbon returns to the food chain [1]. Here, we propose the soil biological loop as an integrated framework. Within this framework, higher trophic organisms interact with viruses and microorganisms to modulate biogeochemical processes and carbon fluxes, thereby governing soil carbon sequestration. Interactions within this biological loop are expected to influence SOM formation and stabilization by regulating microbial biomass production and microbial necromass accumulation. Microorganisms are subject to predation by fauna and protists, as well as viral lysis—key pathways of microbial growth and death within the biological loop. The microbial carbon pump (MCP) refers to the process by which soil microbes metabolize plant-derived photosynthates and translocate the resulting carbon into the soil via biosynthesis, metabolism, and necromass generation [2]. To assess the impact of the biological loop on the global soil organic carbon (SOC) pool, it is crucial to understand how its interactions influence SOC formation and stabilization via MCP.

In this perspective, we explore how components of the biological loop interact and modulate carbon sequestration dynamics by regulating soil food web structure and necromass formation (Fig. 1). To date, most studies on soil carbon sequestration have focused on mechanisms underlying the MCP and biological carbon pump (BCP) [2,3]. We further extend these classical frameworks and discuss how the virus-mediated microbial carbon pump (v-MCP) regulates soil carbon dynamics. The v-MCP focuses on the dynamic translocation and transformation of plant-derived resources (i.e. plant litter, photosynthates, and living plant fine roots) across the biological loop under viral infection, ultimately contributing to necromass formation and SOC accumulation. We advocate for integrating the full biological loop, especially the interactions between high trophic biota and viruses, to investigate their impacts on carbon accumulation, thereby offering important insights into the mechanisms underpinning biogeochemical processes under climate change.

**Figure 1.**
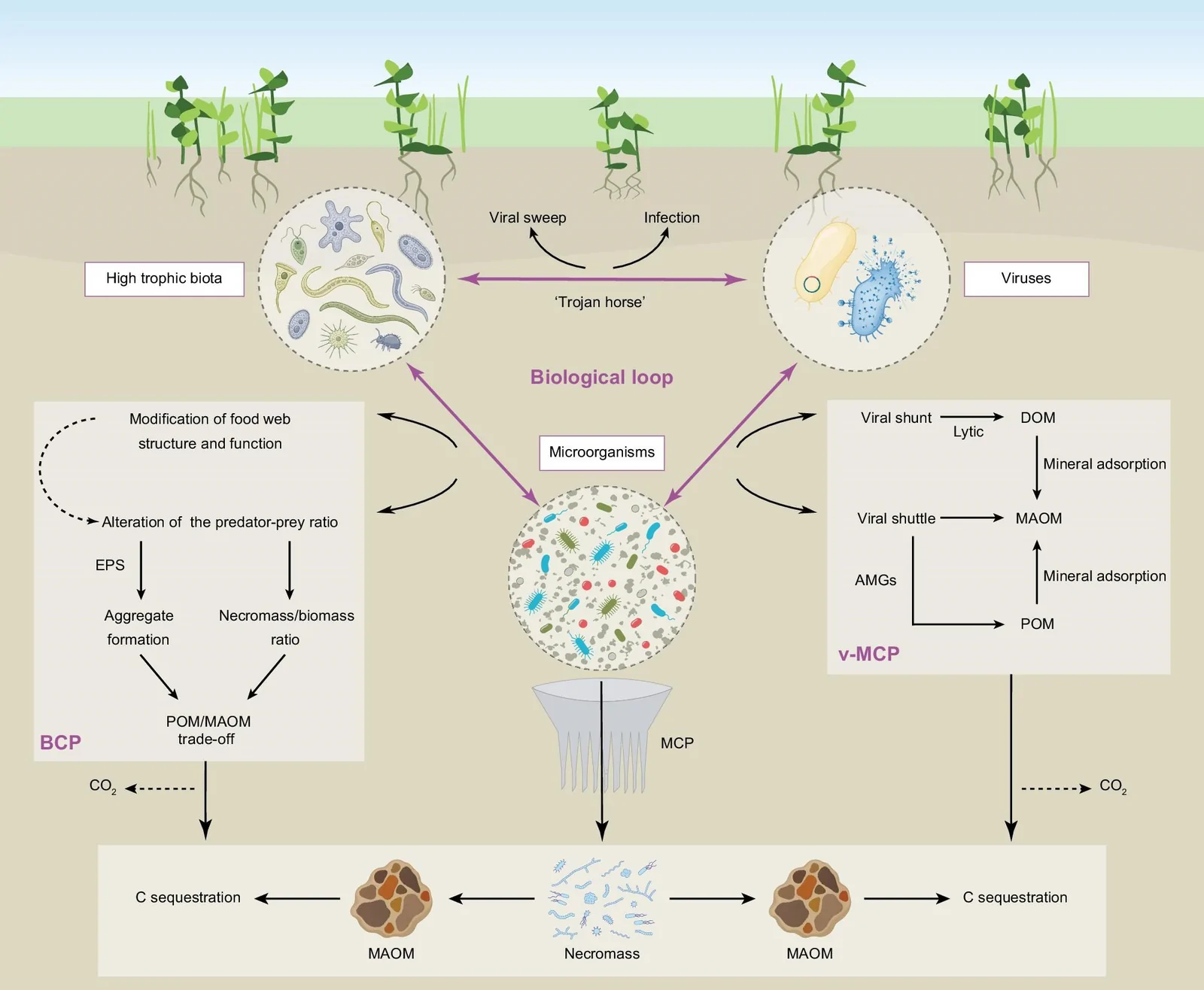
The impacts of the biological loop on soil carbon dynamics. The conceptual framework illustrates how the biological loop components interact and the relationships between high trophic biota, microorganisms and viruses. The MCP framework is supported by existing evidence [2]. The BCP framework highlights the carbon allocation and SOC accrual processes driven by trophic interactions [3]; the v-MCP framework emphasizes the putative influences of viruses on microorganisms and soil carbon sequestration. ‘Trojan horse’ and viral sweep represent the hypothesized impacts of high trophic biota on viruses. EPS, extracellular polymeric substances; DOM, dissolved organic matter; POM, particulate organic matter; MAOM, mineral-associated organic matter; MCP, microbial carbon pump; v-MCP, virus-mediated microbial carbon pump; BCP, biological carbon pump. ‘Trojan horse’: high trophic biota (e.g. protists, invertebrates) harbor soil viruses and facilitate their long-distance horizontal migration within and across soil microhabitats.

**Trophic predation on carbon sequestration.** High trophic biota within the soil food web, such as nematodes and protists, regulate microbial structure and functions through predation (top-down regulation) [3], which mediates soil carbon dynamics and stabilization. High trophic biota predation on microorganisms may influence the turnover of particulate organic carbon (POC) and mineral-associated organic carbon (MAOC), influence the trade-off between these two carbon pools, and thereby emerge as core regulators of soil carbon transformation. A recent study has shown that key functional groups in the soil microbial food web (nematodes, protists, and microorganisms) can explain up to 35% of the variation in CO₂ emissions [4]. High trophic biota also significantly affect the expression of functional genes related to microbial carbon fixation, degradation and growth through resource control and predation [5]. Protists can decrease litter decomposition rates by as much as 40% and increase soil respiration by 17% via shaping microbiome composition [6]. Moreover, the predation by protists is likely to change soil carbon stability by influencing aggregation formation, which indirectly mediate soil carbon dynamics by physical protection. Microbial extracellular polymeric substances (EPS, such as long-chain macromolecular compounds or proteases) are released in response to predation by high trophic biota, and these substances contribute to aggregation formation and SOC protection. Accumulating evidence implies that the interactions between high trophic biota and microorganisms likely contribute to soil carbon accumulation and stabilization by altering food web structure and microbial function.

In addition, high trophic biota alter the formation and stabilization of microbial necromass through the BCP [3], which highlights carbon translocation and transformation driven by trophic interactions across the soil food web. At low predator densities, such as during microbial dispersal to new sites, microbial population growth and activity are enhanced, thereby accelerating the rate of microbial necromass formation. In contrast, higher predator densities exert negative effects on microbial growth and necromass formation. Multitrophic interactions among omnivores-predators, microbivores, and microorganisms substantially modify the ratio of microbial necromass carbon to microbial biomass carbon. Moreover, predation by higher trophic organisms is partly governed by prey body mass; certain protists and nematodes exhibit a preference for smaller microorganisms, thereby shifting the predator–prey ratio. Consequently, larger bacteria and fungi gain a competitive growth advantage, which directly contributes to necromass production and SOC accumulation. Soil faunal grazing on bacteria and fungi governs mineral-associated organic matter (MAOM) formation through microbial pathways. Predation reshapes the ratio of Gram-negative to Gram-positive bacteria. Given their divergent mineral sorption abilities, this shift directly alters MAOM accumulation. Predation also modifies plant root exudation and the composition of predator excreta, facilitating MAOM formation via mineral binding and aggregate development. Furthermore, soil fauna exert divergent controls over arbuscular mycorrhizal (AM) and ectomycorrhizal fungi, which reshape organic matter decomposition [7]. Soil dominated by AM fungi supports higher MAOM accumulation, ultimately affecting the long-term SOC stability.

**Viral infection on carbon accumulation.** As important components of biota communities, viruses regulate host population dynamics and microbial evolution. Increasing attention has been paid to the effects of virus–microbe interactions on carbon accumulation. Viruses mediate the marine carbon cycle primarily through two mechanisms—the viral shunt and viral shuttle—to drive the vertical flux of carbon [8,9]. The viral shunt converts carbon in microbial biomass from particulate organic carbon into predominantly dissolved lysates, part of which is utilized by the active microbial community, delaying downward carbon export [8]. The viral shuttle hypothesis posits that the release of ‘sticky’ organic polymers during lysis promotes particle aggregation and association with minerals, facilitating carbon sequestration [9]. However, the viral shunt and viral shuttle established for marine environments are not universally applicable to carbon cycling dynamics in soil, owing to the unique physical nature of soils such as variable aggregate and pore structures.

Viral infection of host cells occurs in soils via two main strategies: lytic infection and lysogenic infection. During lytic infection, viruses induce host lysis through top-down control, accompanied by the release of cytosolic compounds and necromass in soils. These cytosolic compounds can be recycled by the surviving microorganisms via the viral shunt. Consequently, the resulting bloom of new microbial growth likely promotes necromass production and the formation of persistent carbon compounds through MCP. The viral shunt channels organic compounds back into the biological loop, thereby regulating carbon and nutrient cycling as well as energy transfer in soils. In contrast, lysogenic infection involves the integration of the viral genome into host genomes, thus regulating host metabolic pathways. Released dissolved organic carbon can be stabilized via mineral binding and physical protection by soil aggregates. Lysogenic viral lifestyles facilitate the expression of auxiliary metabolic genes (AMGs), which remodel metabolic pathways in host microbes [10]. This metabolic reprogramming likely promotes the formation of aggregate-occluded particulate organic matter and MAOM [10]. These two physically protected carbon fractions are critical for long-term SOC sequestration. Furthermore, viruses may impact microbial community dynamics and competition, as well as carbon use efficiency, thereby altering the carbon allocation between biomass and respiration. These interactions further influence the carbon entombing effect of the MCP.

Based on the above, we propose the term ‘virus-mediated microbial carbon pump’ (v-MCP) to describe the microbial entombing effect and priming effect on soil carbon dynamics driven by viruses. The v-MCP framework emphasizes that, through viral AMGs, viruses modulate both intracellular microbial anabolism (*in vivo* turnover) and the extracellular enzymatic processing of SOC (*ex vivo* modification), thereby altering the stabilization of fresh organic inputs into persistent carbon pools. Viral AMGs encoding carbon-degrading enzymes may enhance organic matter decomposition, labile DOM production, and priming effects, whereas those associated with carbon fixation, polysaccharide biosynthesis, or EPS production may promote microbial biomass formation, aggregation, and carbon retention. Furthermore, viral lysis may enhance the turnover efficiency of the MCP by facilitating the conversion of microbial biomass into necromass.

The mechanisms of viral regulation on soil carbon cycling shift with environmental conditions. Within resource-rich soils, viral predation often stimulates bacterial proliferation and expedites MCP turnover by converting microbial biomass to necromass, ultimately facilitating SOC sequestration. In resource-limited soils, however, viruses reprogram host metabolism to prioritize catabolic respiration over anabolic growth, leading to the consumption rather than synthesis of cellular biomass. Importantly, the net effect of viral AMGs on SOC dynamics is also likely context-dependent. For example, viral AMGs annotated as glycosyltransferases, which are involved in polysaccharide biosynthesis, were enriched in chemical-only fertilized soils compared with organic-only fertilized soils [11]. Thus, whether viral AMGs boost SOC stabilization or carbon loss may depend on the relative dominance of degradative versus biosynthetic functions, together with soil resource availability and mineral protection capacity.

Despite these observations, empirical research regarding viral regulation over priming and entombing effects in soils with divergent resource status remains scarce, and the relevant mechanisms are largely unresolved. To accurately quantify the effects of lytic and lysogenic viruses on soil carbon, further research is needed to elucidate the biological mechanisms that regulate the switching between viral infection modes, factors influencing v-MCP efficiency, and their impacts on soil carbon dynamics and stability.

**Coupling mechanisms between high-trophic predation and viral infection.** The concept of the multitrophic biological loop vividly illustrates the flow of energy and matter among fauna, protists, microorganisms, and viruses. High trophic biota (e.g. protists) not only graze on bacteria but also directly consume viruses—a process termed virovory—underscoring the complex interactions that go beyond binary grazing versus lysis [11]. Through phagotrophy, high trophic biota can remove viral particles from soil environments, thereby reducing viral abundance. Additionally, the food vacuoles of high trophic biota may serve as a ‘Trojan horse’ for viruses, allowing them to evade harsh environmental stress and facilitating long-distance viral dispersal. Moreover, viral capsid lipid membranes are rich in carbon and energy substrates. Through protist grazing, viral-derived carbon is recycled back into the biological loop, representing a key process defined as the ‘viral sweep’ [12]. For some protist species, viral particles can even serve as a primary source of carbon and nitrogen to meet their nutritional demands. Compared to abiotic processes alone, protists could increase the daily viral removal rate by up to 85% in coastal environments [13]. Despite the well-documented occurrence of the viral sweep in marine ecosystems, whether this process exists in soil environments and its potential contribution to soil carbon cycling remain largely uncharacterized.

Trophic diversity of soil fauna is primarily driven by environmental factors such as land use and climate. Beyond these factors, resource availability (e.g. SOC content) and soil structure (e.g. clay fraction) further regulate the trophic diversity of microbivores. Specifically, SOM acts as a vital substrate for trophic interactions between microbivores and microorganisms, while soil pore structure modulates the accessibility of microbial prey. Under resource-limited conditions, soil fauna enhance niche partitioning and alter feeding strategies. This increases the diversity of bacterial and fungal prey consumed by microbivores, which may trigger the switch of viruses toward the lysogenic cycle within their host and weaken the viral shunt for carbon allocation.

In soils with high clay content, poor habitat permeability restricts foraging by soil fauna. Reduced predation by higher-trophic organisms may also alter viral life strategies and viral shunt processes, thereby ultimately modulating soil carbon dynamics and ecosystem functions. However, empirical evidence regarding how predation pressure modulates the viral shunt process and the expression of viral AMGs via alterations in host density and community composition remains limited. Furthermore, the mechanisms by which predation regulates the transition between lysogenic and lytic viral cycles are still poorly understood. This underscores the need for an integrative perspective centered on multitrophic interaction networks. Conversely, viral infection can feed back to high-trophic predators by reshaping the resource base and prey landscape. Lytic infection reduces the abundance of susceptible microbial prey while releasing labile DOM that can stimulate the growth of resistant or opportunistic microbial populations, thereby altering prey availability and quality for microbivores. Lysogenic infection may further modify host traits, including growth rate, cell-surface properties, stress tolerance, and metabolic activity, which can change the vulnerability and nutritional value of microbial prey. These virus-driven shifts in microbial community composition and host physiology are likely to affect the feeding efficiency of protists and soil fauna, creating reciprocal feedbacks between viral infection and trophic predation within the biological loop.

We further hypothesize that (i) increased grazing pressure will reduce susceptible host density, favor more defended or stress-tolerant microbial populations, and shift viral infection toward lysogeny, thereby weakening carbon release mediated by the viral shunt; and (ii) relaxed predation or resource-rich conditions may favor lytic infection, increase labile DOM release, stimulate opportunistic microbial growth, and raise prey availability for microbivores. These hypotheses can be tested using microcosms that manipulate predator abundance and viral activity, combined with stable isotope tracing, metagenomics, metatranscriptomics, and measurements of DOM, microbial necromass, MAOC, and CO₂ production. Notably, predation and viral lysis represent fundamentally different pathways of microbial mortality. Predation primarily influences the production of particulate organic carbon, whereas viral lysis mainly generates dissolved organic carbon through cellular lysis. It remains unclear whether these two carbon products differ systematically in their chemical composition, mineral association potential, and turnover time and how these discrepancies ultimately affect long-term SOC stabilization.

**Future perspectives and challenges.** Soil fauna such as termites, ants, and earthworms also drive ecosystem engineering processes in soils, thereby strongly regulating the terrestrial biological loop via biogenic structure construction [14]. By altering soil physical properties such as pore structure and moisture content, these soil invertebrates further modify microbial activity and nutrient availability, thereby establishing interconnected biological cycles [14]. Collectively, soil fauna bridge soil nutrients, microorganisms and plants to sustain carbon turnover, acting as indispensable components that maintain the stability of the global terrestrial biological loop.

Dynamic shifts in soil physical structure (e.g. soil texture and aggregate architecture) driven by soil fauna exert pronounced controls over interactions within the biological loop. For example, fine-textured clay soils restrict large predators such as nematodes from accessing microbial prey, while smaller predators, including viruses, remain capable of reaching their targets. Additionally, soil fauna serve as viral vectors to facilitate virus translocation across soil matrices [15]. Earthworm bioturbation reshapes soil architecture and generates biopores, which act as preferential flow pathways and accelerate viral dispersal. Furthermore, intestinal viruses harbored by soil fauna reveal a unique pattern of fauna–virus interaction within the biological loop. Further research should focus on elucidating the influences of fauna–virus interactions on SOC accrual.

Despite the recognized importance of high trophic biota and viruses, they remain rarely integrated into biogeochemical models due to several challenges. A major obstacle is the vast ‘dark matter’ of uncultivated fauna and viruses, whose activities *in situ* remain a black box. High trophic biota exhibit enormous functional diversity, with species-specific feeding strategies, prey preferences, and environmental sensitivities. A functional trait-based framework, which classifies protists and nematodes by ecological role rather than taxonomy, offers a promising solution. Likewise, viruses display divergent life strategies (lytic versus lysogenic), resulting in highly complex, context-dependent impacts on carbon cycling. Accordingly, viral life strategies must be explicitly incorporated into models.

Multitrophic biological loop interactions are inherently dynamic and shift with environmental conditions. Mechanistic models capable of capturing this complexity are critical for predicting ecosystem responses to global change. Emerging approaches such as nanoscale secondary ion mass spectrometry (nanoSIMS) coupled with stable isotope tracing offer great potential to disentangle multitrophic interactions within the biological loop and track virus-induced metabolic responses in host cells or microbial hotspots. Addressing these challenges is essential for accurately predicting ecosystem functioning under the dual pressures of climate change and biodiversity loss.
